# Incidence and Determinants of Acute Kidney Injury after Prone Positioning in Severe COVID-19 Acute Respiratory Distress Syndrome

**DOI:** 10.3390/healthcare11212903

**Published:** 2023-11-04

**Authors:** Riccardo La Rosa, Benedetta Grechi, Riccardo Ragazzi, Valentina Alvisi, Giacomo Montanari, Elisabetta Marangoni, Carlo Alberto Volta, Savino Spadaro, Gaetano Scaramuzzo

**Affiliations:** 1 Department of Translational Medicine and for Romagna, University of Ferrara, 44124 Ferrara, Italy; riccardo.larosa@edu.unife.it (R.L.R.); benedetta.grechi@edu.unife.it (B.G.); riccardo.ragazzi@unife.it (R.R.); vlc@unife.it (C.A.V.); savino.spadaro@unife.it (S.S.); 2 Anesthesia and Intensive Care Unit, Emergency Department, Azienda Ospedaliera Universitaria Ferrara, 44124 Ferrara, Italy; v.alvisi@ospfe.it (V.A.); g.montanari@ospfe.it (G.M.); e.marangoni@ospfe.it (E.M.)

**Keywords:** acute respiratory distress syndrome, acute kidney injury, prone positioning, mechanical ventilation, central venous pressure

## Abstract

(1) Background: Acute kidney injury (AKI) is common among critically ill COVID-19 patients, but its temporal association with prone positioning (PP) is still unknown, and no data exist on the possibility of predicting PP-associated AKI from bedside clinical variables. (2) Methods: We analyzed data from 93 COVID-19-related ARDS patients who underwent invasive mechanical ventilation (IMV) and at least one PP cycle. We collected hemodynamic variables, respiratory mechanics, and circulating biomarkers before, during, and after the first PP cycle. PP-associated AKI (PP-AKI) was defined as AKI diagnosed any time from the start of PP to 48 h after returning to the supine position. A *t*-test for independent samples was used to test for the differences between groups, while binomial logistical regression was performed to assess variables independently associated with PP-associated AKI. (3) Results: A total of 48/93 (52%) patients developed PP-AKI, with a median onset at 24 [13.5–44.5] hours after starting PP. No significant differences in demographic characteristics between groups were found. Before starting the first PP cycle, patients who developed PP-AKI had a significantly lower cumulative fluid balance (CFB), even when normalized for body weight (*p* = 0.006). Central venous pressure (CVP) values, measured before the first PP (OR 0.803, 95% CI [0.684–0.942], *p* = 0.007), as well as BMI (OR 1.153, 95% CI = [1.013–1.313], *p* = 0.031), were independently associated with the development of PP-AKI. In the multivariable regression analysis, a lower CVP before the first PP cycle was independently associated with ventilator-free days (OR 0.271, 95% CI [0.123–0.936], *p* = 0.011) and with ICU mortality (OR:0.831, 95% CI [0.699–0.989], *p* = 0.037). (4) Conclusions: Acute kidney injury occurs frequently in invasively ventilated severe COVID-19 ARDS patients undergoing their first prone positioning cycle. Higher BMI and lower CVP before PP are independently associated with the occurrence of AKI during prone positioning.

## 1. Introduction

Acute kidney injury (AKI) is common in COVID-19 patients, with its prevalence often exceeding 25% in critically ill patients [1]. Both systemic and local mechanisms, such as endothelial dysfunction, direct viral damage, and hemodynamic disturbances, may converge on kidney injury in COVID-19-related AKI [1,2,3]. In critically ill patients, COVID-19-related AKI shows an overwhelming prevalence of progression, and renal replacement therapy (RRT) is required in more than 30% of cases [4].

Prone positioning (PP) has been extensively used in the management of severe COVID-19 ARDS (C-ARDS) [5] for its beneficial effects on V/Q mismatch, gas exchanges, and respiratory mechanics [6,7,8]. The rationale behind PP lies in improved regional ventilation homogeneity [9] and the enhancement of a lung-protective ventilation strategy [10,11]. Despite large trials highlighting the positive impacts of PP in patient outcomes, including mortality [12], PP is not free from potential adverse effects, like procedural risks, loss of venous access points, involuntary extubation, and potential hemodynamic instability [10,13]. Moreover, heterogeneous responses to prone positioning have been found among C-ARDS patients, suggesting the possibility that not all patients may equally benefit from this procedure [14].

Pathophysiological insights on how PP affects hemodynamics and organ perfusion are lacking. Some evidence has shown that PP may increase cardiac output and help with right ventricle unloading [15]. Additionally, PP may improve hypercapnia, hypoxemia, and intrathoracic driving pressures, ultimately reducing the stress on right ventricular function and improving hemodynamics [10,15]. On the other hand, PP increases intra-abdominal pressure (IAP) [16] and can thus potentially affect abdominal organ perfusion and kidney function [16,17]. Considering its potential role in increasing IAP, prone positioning may therefore be an adjunctive factor in the development of AKI. Nevertheless, the incidence of AKI in patients undergoing prone positioning is still not known. 

In this context, we aimed to assess the incidence of AKI in patients affected by COVID-19 ARDS and undergoing their first prone positioning cycle. Furthermore, we tested if PP-AKI could be associated with demographic, hemodynamic, and clinical factors, which were measured before PP.

## 2. Materials and Methods

### 2.1. Study Design

This is a monocentric retrospective cohort study. The data herein refer to patients admitted to the mixed medical–surgical Intensive Care Unit of Arcispedale Sant’Anna (Ferrara, Italy). Referring to the pandemic outbreaks in our territory, we analyzed data for all adult patients admitted to the ICU between 8 March 2020 and 31 December 2021. In our analysis, we screened all patients admitted to the ICU during the study period for inclusion and included patients who met the following criteria: (a) admission to the ICU for acute respiratory failure due to COVID-19 pneumonia, as assessed through positive RT-PCR tests on nasopharyngeal or endotracheal samples; (b) need for endotracheal tube (ETT) placement during the ICU stay (or one already present at ICU admission); (c) and execution of at least one PP cycle during controlled mechanical ventilation, as ordered by the treating physician and according to available guidelines at the time of the patient’s ICU stay [18]. We excluded patients with the following characteristics: (a) post-surgical patients; (b) patients admitted for medical reasons other than COVID-19 pneumonia; (c) patients who already had an AKI diagnosis at the time of their ICU admission or in the previous three months; (d) and patients with end-stage CKD, in line with recent studies on AKI [19]. If patients were readmitted to the ICU, only the first admission was considered for analysis. We collected patient-level data through the digital records of their hospital stays. Patients whose digital record of their ICU stay was incomplete and/or unavailable for data collection were excluded.

The study protocol received approval from the local ethical committee (approval number 544/2022/Oss/AOUFe) and adhered to the Declaration of Helsinki. In accordance with local regulations, informed consent was collected or, if collection was not possible, waived.

### 2.2. Definitions

For this study, the definition and classification of acute kidney injury were made according to the latest KDIGO Guidelines [20]. As per clinical practice, all enrolled patients had an indwelling urinary catheter during their ICU stay and had their urinary output monitored hourly and daily serum creatinine (SCr) measurements taken. This allowed for the retrospective diagnosis of AKI by monitoring both any decrease in urinary output and any daily increase in SCr. We defined PP-associated AKI (PP-AKI) when AKI onset occurred at any time between the start of the PP cycle and the 48 h after returning to the supine position. The 48 h cutoff was chosen because of the slow kinetics of serum creatinine after renal injury [21] and according to the KDIGO criteria for acute kidney injury, in which a wide time range (i.e., up to 48 h) is needed to detect stage III AKI when considering urinary output [22].

### 2.3. Data Collection

We evaluated the following clinical variables during the ICU stay: age, sex, weight, Simplified Acute Physiology Score II (SAPS-II) [23], length of ICU stay, and ICU survival. Baseline SCr was defined as the most recent value within 30 days before the ICU admission. Other baseline data collected included: hemodynamic data (HR, arterial pressure); PaO_2_/FiO_2_ ratio at ICU admission; serum lactates at ICU admission; and hemoglobin (Hgb) and hematocrit (Hct) at ICU admission. We also collected the following variables before the initiation of PP: SCr the day of PP; total urinary output 24 h before PP; cumulative fluid balance (CFB) since ICU admission; hemodynamic data (SAP, central venous pressure (CVP), HR); and circulating biomarkers (i.e., Hgb, Hct, serum lactates, BUN). Specifically, the value of central venous pressure was collected in mechanically ventilated patients before entering the prone position and while they were in the semi-recumbent position.

The infusion of diuretic and/or vasoactive drugs before PP was recorded, along with their daily dosage. Hourly urinary output and evolution in SCr during PP cycle and until 48 h after returning to the supine position permitted the diagnosis of AKI according to KDIGO criteria. 

In the interval between the beginning of PP and 48 hours after returning to the supine position, we registered any introduction or increase in dosage for both diuretic(s) and vasoactive drugs. Lactates and CFB were monitored during the same interval.

### 2.4. Study Aims

This trial was designed to assess the incidence of AKI and its possible determinants in a population of severe COVID-19 ARDS patients undergoing prone positioning for the first time. Our primary aim was to define the incidence and characteristics of PP-AKI in the analyzed population. Our secondary aims were as follows: (a) to determine if there were significant differences in clinical variables between patients who developed PP-AKI after the first PP and patients who did not; (b) to determine if any of the clinical variables preceding prone positioning could have a predictive value on AKI onset; (c) and to evaluate association of clinical variables preceding the first PP cycle with ICU mortality and/or ventilator-free days (VFDs).

### 2.5. Statistical Analyses

Standard descriptive analysis was performed to outline the features of the overall population and characteristics of each defined group. Patients were divided into two groups according to the development of PP-related AKI. Data are displayed as mean (standard deviation) for normally distributed variables; otherwise, they are presented as median [IQ range]. The Shapiro–Wilk test and Kolmogorov–Smirnov tests were used to determine if the variables were normally distributed. Two tailed *t*-tests or the Mann–Whitney U-test were performed on independent samples as appropriate, allowing for a comparison between clinical variables and the detection of any significant inter-group differences.

Binomial logistical regression was performed to identify any predictive value of clinical variables on PP-AKI onset and ICU mortality. Multivariable linear regression was used to identify predictors of VFDs. For the logistic regression on the predictors for PP-AKI onset, we decided to include CVP and SCr, both measured before the first PP, and the 24 h CFB/weight ratio before PP. The model was adjusted using the following variables: age, BMI, SAPS-II score before PP, presence of previously known chronic kidney disease (CKD), and maximal positive end-expiratory pressure (PEEP) applied during PP. Duration of PP cycle and duration of hospital stay before ICU admission were also included. This modeling approach was designed to prioritize the choice of clinically relevant variables. For the choice of variables selected for the other two aforementioned models, more details are available in the Supplementary Materials.

A Variance Inflation Factor (VIF) > 5 was considered as the cut-off for collinearity between variables. Finally, receiver operating characteristic (ROC) curves were built to ascertain predictive performance and optimal cut-offs for variables correlating with AKI development.

Sample size was calculated based on the primary outcome, i.e., the incidence of AKI in patients undergoing PP. Considering an expected prevalence of 30% and a precision of 0.1, we calculated a required sample size of 81 patients at 95% CI and 10% margin of error. Considering a dropout rate of 10%, a sample size of a minimum of 89 patients was required [24]. All analyses were performed using statistical analysis software (SPSS ver. 26.0; IBM Corp., Armonk, NY, USA), and the graphical elaboration of data was carried out using GraphPad software (Prism ver. 9.4; GraphPad Software, San Diego, CA, USA). As a conventional criterion, *p*-values < 0.05 were used to assess statistical significance.

## 3. Results

### 3.1. Population

**Figure 1:**
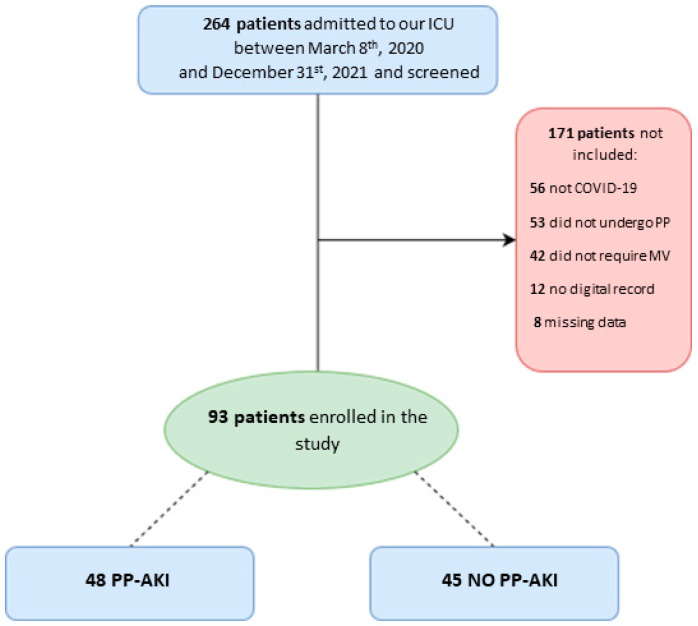
Patient flow diagram for the present study. ICU = intensive care unit; PP = prone positioning; MV = mechanical ventilation; PP-AKI = acute kidney injury temporally related to prone-position.

We screened 264 patients admitted to our ICU during the study period. Among them, 56 were not affected by SARS-CoV-2 pneumonia, 42 did not undergo invasive mechanical ventilation, and 53 did not undergo prone positioning; we were not able to retrieve digital records for 12 patients, while for 8 patients, a large amount of data were missing. In the end, data from 93 patients were analyzed (Figure 1).

The demographic characteristics of the study population are reported in Table 1. A total of 75/93 (81%) were males, with a median PaO_2_/FiO_2_ at ICU admission of 105 [79–133] mmHg. The median age was 67 [59–72.5] years, and their ICU stay began at 2 [1–4] days since hospital admission. The median BMI was 29.5 [26.5–33.6] kg/m^2^. The most frequent comorbidity was arterial hypertension, which affected 48/93 (52%) subjects. Prone positioning was maintained for a median time of 39 [24–47] hours. In the overall population, the median number of completed PP cycles (i.e., pronation and return to the supine position) were two [1–3], with 8/93 (8%) patients subjected to four PP cycles and only 6/93 (6%) patients completing five cycles.

### 3.2. Incidence and Characteristics of PP-Associated AKI

A total of 48/93 (52%) patients developed AKI during or in the 48 h after the first prone positioning cycle (Table 2). The main source for diagnosis was urine output, which was diagnostic in 24/48 (50%) of patients. Creatinine elevation alone was diagnostic in 10/48 (21%) cases, whereas urine output and SCr were both diagnostic in 14/48 (29%) of patients. 

**Figure 2:**
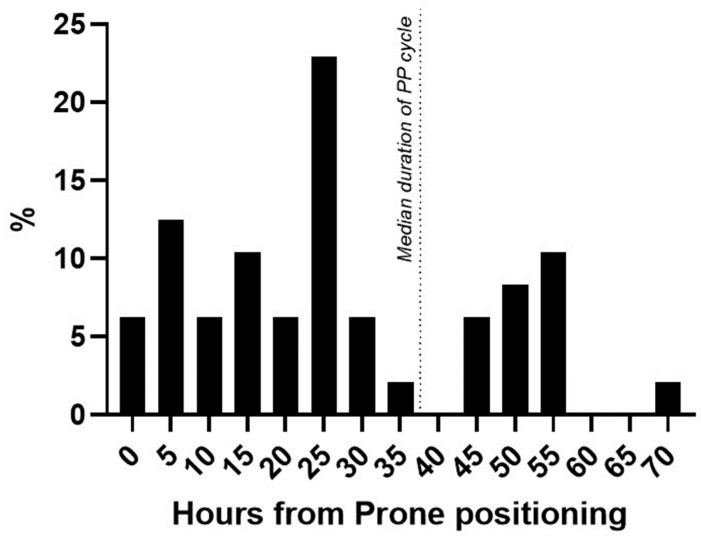
Time of AKI diagnosis from PP start. Percentage distribution of patients newly diagnosed with AKI from the start of prone positioning. Dotted line = median duration of prone positioning in the AKI cohort.

Regarding the KDIGO classes, 29/48 (60%) patients were diagnosed as class I AKI, 12/48 patients (25%) as class II, and 7/48 patients (15%) as class III AKI. Among patients that developed AKI after first PP, 11/48 (23%) needed RRT during ICU stay, while 4/45 (9%) of patients did not develop PP-AKI (*p* = 0.07). Median PP-AKI onset was at 24 [13.5–44.5] hours after starting prone positioning (Figure 2).

Patients diagnosed by urinary output were diagnosed earlier (22.4 [15.8–28.9] hours) compared to patients diagnosed using the Scr value (42.3 [28.1–56.5] hours, *p* = 0.003).

Among the PP-AKI patients, 27/48 (56%) had furosemide infusion before PP, with a median dosage of 60 [40–80] mg/24 h. However, furosemide dosage before pronation did not significantly differ between patients who developed AKI and patients who did not (ꭓ^2^ = 6.07, *p* = 0.41).

### 3.3. Differences between the AKI and Non-AKI Populations

Differences between the groups are detailed in Table 3. There were no significant differences in age (68 [61–74] vs. 66.5 [58.5–72] years, *p* = 0.53), SAPS-II score (33 ± 6 vs. 34 ± 7, *p* = 0.28), and PaO_2_/FiO_2_ (107 [79–131] vs. 102.5 [80–133], *p* = 1) at ICU admission. Urinary output was not significantly different between the groups in the 24 h before starting PP, and median SCr before PP was within normal range in both groups, although PP-AKI patients had a slightly more elevated SCr before the start of the PP cycle (Table 3). Before starting the first PP cycle, patients who developed PP-AKI had a significantly lower cumulative fluid balance, even when normalized for body weight (21.4 [6.5–42.8] vs. 4.4 [0–24.7] mL/kg, *p* = 0.007). PP-AKI patients also tended to present a lower CVP, but this difference did not reach statistical significance (*p* = 0.16). Moreover, the mean duration of the first PP cycle was similar in the two groups, as were the median ventilation settings (tidal volume, PEEP) and infusion of diuretic drugs (i.e., furosemide). Comparing clinical outcomes, no significant differences were found in terms of ICU length of stay (considering only survivors), number of PP cycles, ventilator-free days (VFD), or in ICU mortality (Table 3).

### 3.4. Determinants of PP-AKI

To evaluate the independent determinants of PP-AKI, we performed a binomial logistical regression analysis (Table 4). Our model was statistically significant in predicting PP-AKI (χ^2^ = 24.8; *p* = 0.02). CVP measured before the first PP (OR = 0.80, CI [0.68–0.94], *p* = 0.007) and BMI (OR = 1.15, CI [1.01–1.31], *p* = 0.03) were independently associated with the development of PP-AKI.

### 3.5. Predictors with Mortality and VFDs

We performed a multivariable linear regression analysis with VFDs as the dependent variable. The model was significant (*p* = 0.001), and the CVP before the first PP cycle showed a correlation with the number of VFDs (OR = 0.27, CI [0.12–0.93], *p* = 0.01). On the contrary, a higher number of days of ICU stay before first PP, a higher number of PP cycles, and the presence of previous CKD were all negatively correlated with the number of VFDs.

A higher BMI showed a borderline significant contribution, according to the model (OR = −0.19, CI [−0.59–0.037], *p* = 0.08). Furthermore, binomial logistical regression, with ICU mortality as dependent variable, was performed and showed that an independent correlation between a lower value of CVP before the first PP cycle and ICU mortality (OR = 0.83, CI [0.69–0.98], *p* = 0.004) (χ^2^ = 36.6; *p* < 0.001)). A higher number of days in the ICU before the first PP cycle (OR = 1.00, CI [1.00–1.00], *p* = 0.003) and a higher number of PP cycles correlated (OR = 2.82, CI [1.41–5.62], *p* = 0.003) with mortality.

## 4. Discussion

We found that more than half of patients undergoing their first prone positioning cycle for severe COVID-19 ARDS developed AKI during or immediately after the prone positioning cycle. Indirect signs of hypovolemia, such as lower central venous pressure, cumulative fluid balance, and BMI, were significantly associated with the diagnosis of AKI related to prone positioning.

Acute kidney injury is common in patients affected by COVID-19 [4], and its pathophysiology is multifactorial. Similarly to sepsis-related kidney injury, regional and systemic inflammation can contribute to vascular and hemodynamic alterations, leading to organ damage [2]. Moreover, in patients with C-ARDS, hypoxia and right-sided heart dysfunction may further exacerbate kidney injury [2].

Prone positioning can potentially increase the risk of AKI by increasing the intra-abdominal pressure (IAP) [16]. The link between increased IAP and AKI has been already explored in patients with intra-abdominal hypertension, showing an increased risk for AKI [25,26]. It is also known that PP increases in IAP may compromise abdominal organ perfusion, potentially damaging kidney function by reducing the renal fraction of cardiac output and increasing renal vascular resistances [16,17].

Prone positioning showed great applicability in the recent COVID-19 pandemic, but there are no data on its temporal association with the diagnosis of AKI. In our study, we found that these patients have a high incidence of AKI diagnosis (more than half of patients receiving diagnosis) during the first PP cycle. Moreover, we found that indirect indexes of hypovolemia, such as low CVP and lower cumulative fluid balance, increased the risk of PP-AKI independently.

The central venous pressure is a relatively simple index that is obtainable at bedside and has a good correlation with preload and volume status [27,28]. Abdominal organ perfusion, including the kidneys, may be affected by variations in CVP. Indeed, kidney perfusion is physiologically driven by the difference between renal arterial and venous pressure; a rise in the latter may depend on CVP elevation, thus reducing net kidney perfusion [29]. Excessive levels of CVP can endanger critically ill patients, as large datasets suggest [30,31]. On the other hand, very low values of CVP suggest a low-volume state and possibly a good tolerance to fluid administration [32]. Optimizing volume status and cardiac output may thus improve organ perfusion and provide a protective strategy against kidney damage [2]. In fact, fluid restriction and diuretic drugs are widely used in acute lung injury management, including C-ARDS to prevent further deterioration in lung function [33,34]. Indeed, hypovolemia was a frequent finding in our retrospective cohort, and patients who developed PP-AKI had a significantly lower weight-normalized cumulative fluid balance and a lower CVP before starting their first PP cycle. The novelty of our findings lies in the potential that lower CVP is a predictor of AKI. Indeed, as higher CVP is already known to be associated with kidney damage [29,31], lower values suggest that hypovolemia may play a key role in exacerbating AKI in the context of prone positioning, along with other pathological pathways.

We found that also BMI independently increased the risk of PP-AKI. This confirms what has previously been found in the overall critically ill population, independent from PP [17]. However, in the clinical scenario of prone positioning, BMI may exert an influence on intra-abdominal pressure by further reducing abdominal compliance and more markedly increasing IAP when prone [25].

**Figure 3:**
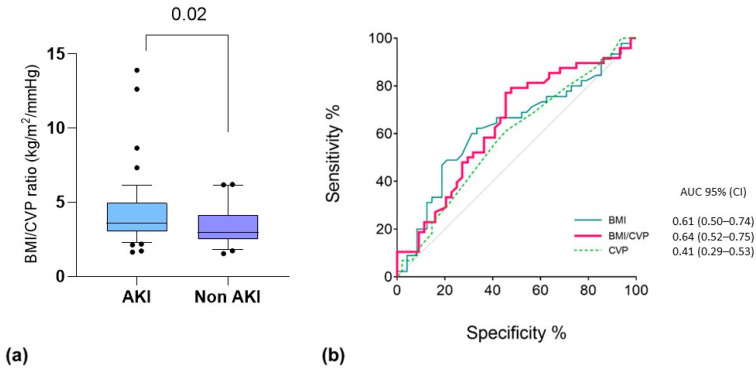
(**a**) Comparative test for BMI/CVP ratio (kg/m^2^/mmHg) between patients who developed AKI associated with the first PP cycle. Data are the results of non-parametric Mann–Whitney U tests for the respective groups. Box: 25-75 percentile; whiskers: 10-90 percentile. (**b**) ROC curve analysis for PP-AKI related to the first PP cycle. The variables tested include the following: CVP before the first PP cycle (dashed green line; *p* = 0.16); BMI at baseline (light blue line; *p* = 0.04); BMI/CVP ratio (red line; *p* = 0.02).

Based on the good performance of the single variables (i.e., CVP and BMI) in the multivariable analysis and according to our physiological interpretation of the results (i.e., a higher impact of PP, as well as IAP elevation, in hypovolemic obese patients), we decided to combine the two predictors into one variable. Thus, we integrated both CVP and BMI into a new index, calculated as BMI/CVP (Figure 3). We found that this index was able to provide a prediction of patients who were going to experience AKI after their first prone positioning cycle with moderate accuracy by integrating the effect of hypovolemia on kidney perfusion and that of BMI on IAP. This should be considered exploratory since it was not a predefined end-point of the study.

Prone positioning has extensively demonstrated a survival benefit in moderate–severe ARDS patients [5,6,7,8,9], despite the possible effect on intrabdominal pressure. In our study, we did not test if PP can increase the risk of AKI, as this is out of the scope of the current study. Moreover, our findings do not aim to reduce the clinical importance of prone positioning but to underline that the incidence of AKI diagnosed during PP in severe COVID-19 patients is high (>50%), especially when performing prolonged PP, and that clinical variables measured before PP may predict this event. Whether the correction of these factors (i.e., hypovolemia) may reduce the occurrence of PP-AKI must be explored in future studies.

To date, our study is the first to investigate the occurrence of AKI in patients undergoing prolonged prone positioning. Previous studies have suggested that PP may represent a trigger for the elevation of IAP [16,35] and impaired venous return and kidney function, especially when hemodynamic stability and volume status are already compromised [35,36]. Moreover, a recent letter published by Wintjens and colleagues [37] shows interesting data regarding transient decreases in glomerular filtration in ARDS patients undergoing prone positioning, along with an increase in serum creatinine that subsides a few days after returning to the supine position. These preliminary results may confirm our findings and the hypothesis that volume depletion and lower kidney perfusion may be mechanisms of AKI that prone positioning may intensify over time.

Nevertheless, this study has several limitations. First, this study primarily consisted of a retrospective analysis on a limited sample, and biases related to this kind of study may apply. Moreover, the predictive value of indexes such as CVP and BMI on AKI development was referred to the first PP cycle. Evaluation of the first PP cycle may actually be representative of the pathophysiological changes during prone positioning, as the first PP cycle has also been shown to be independently related to ICU survival and the duration of mechanical ventilation [38]. Another important limitation exists regarding the choice to study a cohort of patients affected by COVID-19 severe ARDS. The pandemic has indeed offered a chance to study the physiologic changes during prolonged prone positioning. However, for this study, we did not evaluate the impact of COVID-19 on the observed phenomena. Further studies involving moderate–severe ARDS patients undergoing prolonged prone positioning should be carried to verify if the etiology of ARDS may impact the occurrence of AKI.

Furthermore, data on IAP both during supine and prone positioning and advanced hemodynamic monitoring data were not available for this retrospective study. This is mainly due to the limited resources available in our ICU during the COVID-19 pandemic, as well as the relative hemodynamic stability of our cohort of patients and therefore for the absence of indications for advanced hemodynamic monitoring. The same reasons apply to the absence of thoraco-pelvic supports in obese patients, meaning that an investigation of the effect of abdominal supports on CVP and AKI was not possible. Larger prospective studies are required to further explore the relation between prone positioning, intra-abdominal pressure, volume status, and AKI onset in patients undergoing prolonged PP cycles during ARDS of any origin. Finally, we did not record the vaccination status and/or status of our patients and therefore cannot make any conclusions about its possible effect on the outcome of the current investigation.

## 5. Conclusions

Acute kidney injury is common in COVID-19 ARDS patients undergoing their first prone positioning cycle. High BMI and low CVP were independently associated with the occurrence of AKI during prone positioning, indicating the possible influencing role of hypovolemia and intra-abdominal pressure on its pathogenesis.

## Figures and Tables

**Table 1:** Demographic characteristics at ICU admission and outcomes during ICU stay.

Variable	
Age (years)	67 [59–72.5]
Males (%)	75/93 (81%)
Arterial hypertension (%)	48/93 (52%)
Ischemic heart disease (%)	7/93 (7%)
Asthma (%)	8/93 (8%)
COPD (%)	2/93 (2%)
Diabetes (%)	14/93 (15%)
Previous use of diuretics (%)	5/93 (5%)
Chronic kidney disease (%)	4/93 (4%)
BMI (kg/cm^2^)	29.4 [26.5–33.62]
Creatinine at ICU admission (mg/dL)	0.89 [0.71–1.14]
Systolic arterial pressure (mmHg) *	130 [120–150]
Diastolic arterial pressure (mmHg) *	75 [60–80]
Heart rate (beats/min) *	80 [70–90]
PaO_2_/FiO_2_ *	105 [79–133]
Arterial lactate * (mmol/L)	1.2 [0.9–1.5]
Hemoglobin (g/dL) *	13.6 [12.4–14.45]
Number of PP Cycles	2 [1–3]
Highest creatinine during ICU stay	1.47 [0.965–3.19]
Ventilator-free days (days)	0 [0–12]
ICU mortality	54/93 (58%)
Hospital days before ICU (days)	2 [1–4]
Days of ICU before first prone positioning	4 [1–7]

* at ICU admission; COPD = chronic obstructive pulmonary disease; BMI = body mass index; ICU = intensive care unit; PP = prone positioning. Median [IQR] or frequency.

**Table 2:** Characteristics of AKI diagnosis after prone positioning.

Variable	
Patients developing AKI after PP	48/93 (52%)
AKI diagnosed using Urine Output	24/48 (50%)
AKI diagnosed using Creatinine	10/48 (21%)
AKI diagnosed using both	14/48 (29%)
KDIGO class 1	29/48 (60%)
KDIGO class 2	12/48 (25%)
KDIGO class 3	7/48 (15%)
Time from PP start to diagnosis (hours)	24 [13.5–44.5]
Furosemide infusion before PP (yes)	27/48 (56%)

AKI = acute kidney injury; KDIGO = Kidney Disease: Improving Global Outcomes; PP = prone positioning. Median [IQR] or frequency.

**Table 3:** Clinical variables during ICU stay in the overall population and in the AKI/non-AKI groups.

Variable	No AKI (45)	AKI (48)	Total (93)
Age (years)	68 [61–74]	66.5 [58.5–72]	67 [59–72]
BMI (kg/m^2^)	27.7 [25.7–32.1]	30.4 [27.7–33.8]	29.4 [26.6–33.6]
Creatinine (mg/dL)	0.85 [0.72–1.02]	0.91 [0.705–1.2]	0.89 [0.71–1.14]
Arterial pressure, diastolic (mmHg)	70 [60–80]	80 [70–82.5]	75 [60–80]
Heart rate (bpm)	80 [70–85]	82.5 [70–97.5]	80 [70–90]
PaO_2_/FiO_2_	107 [79–131]	102.5 [80–133]	105 [79–131]
Arterial lactates (mmol/L)	1.1 [0.9–1.5]	1.2 [1–1.65]	1.2 [0.9–1.5]
Hemoglobin (mg/dL)	13.3 ± 1.8	13.6 ± 1.7	13.5 ± 1.8
SCr before first PP cycle (mg/dL)	0.74 [0.61–0.89]	0.87 [0.68–1.10]	0.82 [0.65–0.98]
Last 24 h urine output (mL)	1742 ± 555	1724 ± 592	1733 ± 570
CFB from ICU entrance (mL)	1615 [644–3770]	413.5 [0–2015]	1107 [0–3016]
CFB from ICU entrance/kg (mL/kg)	21.4 [6.5–42.8]	4.4 [0–24.7]	13 [0–30.2]
SAPS-II score	33 ± 6	34 ± 7	34 ± 6
Hematocrit (%)	36.4 ± 5.7	38.1 ± 4.8	37.3 ± 5.3
Hemoglobin (mg/dL)	11.9 ± 2.2	12.6 ± 1.7	12.2 ± 2
Arterial lactates (mmol/L)	1.5 [1.2–2]	1.45 [1.1–1.9]	1.5 [1.1–2]
Central venous pressure (mmHg)	10 [7–11]	8 [6–10]	9 [6.5–10.5]
PEEP (cmH_2_O)	10 [10–12]	12 [9.5–13]	12 [10–12]
CVP/PEEP ratio	0.83 [0.615–1.1]	0.805 [0.56–1]	0.83 [0.575–1]
Tidal volume (mL)	430 [420–480]	450 [410–480]	450 [420–480]
Duration of cycle (hours)	41 [23–47]	37.5 [24–46]	39 [24–47]
Maximal PEEP (cmH_2_O)	12 [10–12]	12 [10–15]	12 [10–14]
Maximal furosemide dose (mg/24 h)	80 [40–100]	80 [60–160]	80 [60–120]
Worst SCr value (mg/dL)	1.12 [0.89–2.3]	1.84 [1.06–4.12]	1.47 [0.97–3.18]
ICU Mortality	22/45 (49%)	32/48 (67%)	54/93 (58%)
Number of PP cycles	2 [1–2]	2 [1–3]	2 [1–3]
Days ICU (survivors)	26 [17–44]	24.5 [14.2–38.7]	26 [15–40]
VFDs	3 [0–12]	0 [0–13.5]	0 [0–12]

AKI = acute kidney injury; SCr = serum creatinine; PP = prone positioning; PEEP = positive end-expiratory pressure. ICU = intensive care unit; CVP = central venous pressure. Median [IQR] or frequency.

**Table 4:** Logistic multivariable regression analysis on AKI predictors before the first prone positioning cycle.

Variable	OR	95% CI for OR		*p*-Value
Age (in years)	0.951	0.879	1.029	0.214
BMI	1.153	1.013	1.313	0.031
Creatinine before PP	3.346	0.846	13.234	0.085
CVP before PP	0.803	0.684	0.942	0.007
CFB/kg before PP	0.975	0.948	1.003	0.080
SAPS before PP	1.028	0.927	1.140	0.603
Maximal PEEP during PP	0.923	0.777	1.096	0.360
Maximum serum lactate during PP	1.128	0.611	2.084	0.699
Previous story of CKD	0.233	0.008	6.754	0.396
Cycle duration (hours)	0.975	0.934	1.017	0.241
CFB after 48 h from supine positioning	1.000	1.000	1.001	0.032
Days in ICU before 1st PP cycle	1.000	1.000	1.000	0.179
Days in hospital before ICU	1.000	1.000	1.000	0.368
Constant	1.617			0.887

Data refer to the first pronation cycle for all patients. BMI = body mass index; CFB = cumulative fluid balance; SAPS = Simplified Acute Physiology Score; PP = prone positioning; PEEP = positive end-expiratory pressure; CKD = chronic kidney disease; ICU = intensive care unit.

## Data Availability

The dataset used for this study can be made available from the corresponding author upon reasonable request.

## Supplementary Materials

The following supporting information can be downloaded at: https://www.mdpi.com/article/10.3390/healthcare11212903/s1, Table S1: Multivariable regression analysis on predictors of ventilator-free days (VFDs), censored at 28 days; Table S2: Logistic regression analysis on predictors of ICU mortality.

## References

[1] Ostermann M., Lumlertgul N., Forni L.G., Hoste E. (2020). What every Intensivist should know about COVID-19 associated acute kidney injury. J. Crit. Care.

[2] Legrand M., Bell S., Forni L., Joannidis M., Koyner J.L., Liu K., Cantaluppi V. (2021). Pathophysiology of COVID-19-associated acute kidney injury. Nat. Rev. Nephrol..

[3] Zanella A., Florio G., Antonelli M., Bellani G., Berselli A., Bove T., Cabrini L., Carlesso E., Castelli G.P., Cecconi M. (2021). Time course of risk factors associated with mortality of 1260 critically ill patients with COVID-19 admitted to 24 Italian intensive care units. Intensive Care Med..

[4] Lumlertgul N., Pirondini L., Cooney E., Kok W., Gregson J., Camporota L., Lane K., Leach R., Ostermann M. (2021). Acute kidney injury prevalence, progression and long-term outcomes in critically ill patients with COVID-19: A cohort study. Ann. Intensiv. Care.

[5] Alhazzani W., Møller M.H., Arabi Y.M., Loeb M., Gong M.N., Fan E., Oczkowski S., Levy M.M., Derde L., Dzierba A. (2020). Surviving Sepsis Campaign: Guidelines on the management of critically ill adults with Coronavirus Disease 2019 (COVID-19). Intensive Care Med..

[6] Chen Y., Zhang J., Feng H., Wan F., Zhang Y., Tan L. (2021). Prone positioning in intubated and mechanically ventilated patients with SARS-CoV-2. J. Clin. Anesthesia.

[7] Gleissman H., Forsgren A., Andersson E., Lindqvist E., Falck A.L., Cronhjort M., Dahlberg M., Günther M. (2020). Prone positioning in mechanically ventilated patients with severe acute respiratory distress syndrome and coronavirus disease 2019. Acta Anaesthesiol. Scand..

[8] Vetrugno L., Castaldo N., Fantin A., Deana C., Cortegiani A., Longhini F., Forfori F., Cammarota G., Grieco D.L., Isola M. (2022). Ventilatory associated barotrauma in COVID-19 patients: A multicenter observational case control study (COVI-MIX-study). Pulmonology.

[9] Scaramuzzo G., Karbing D.S., Fogagnolo A., Mauri T., Spinelli E., Mari M., Turrini C., Montanaro F., Volta C.A., Rees S.E. (2022). Heterogeneity of Ventilation/Perfusion Mismatch at Different Levels of PEEP and in Respiratory Mechanics Phenotypes of COVID-19 ARDS. Respir. Care.

[10] Guérin C., Albert R.K., Beitler J., Gattinoni L., Jaber S., Marini J.J., Munshi L., Papazian L., Pesenti A., Vieillard-Baron A. (2020). Prone position in ARDS patients: Why, when, how and for whom. Intensiv. Care Med..

[11] Scholten E.L., Beitler J.R., Prisk G.K., Malhotra A. (2017). Treatment of ARDS With Prone Positioning. Chest.

[12] Guérin C., Reignier J., Richard J.-C., Beuret P., Gacouin A., Boulain T., Mercier E., Badet M., Mercat A., Baudin O. (2013). Prone Positioning in Severe Acute Respiratory Distress Syndrome. N. Engl. J. Med..

[13] Taccone P., Pesenti A., Latini R., Polli F., Vagginelli F., Mietto C., Caspani L., Raimondi F., Bordone G., Iapichino G. (2009). Prone Positioning in Patients with Moderate and Severe Acute Respiratory Distress Syndrome: A Randomized Controlled Trial. JAMA.

[14] Cour M., Bussy D., Stevic N., Argaud L., Guérin C. (2021). Differential effects of prone position in COVID-19-related ARDS in low and high recruiters. Intensiv. Care Med..

[15] Jozwiak M., Teboul J.-L., Anguel N., Persichini R., Silva S., Chemla D., Richard C., Monnet X. (2013). Beneficial Hemodynamic Effects of Prone Positioning in Patients with Acute Respiratory Distress Syndrome. Am. J. Respir. Crit. Care Med..

[16] Hering R., Wrigge H., Vorwerk R., Brensing K.A., Schröder S., Zinserling J., Hoeft A., Spiegel T.V., Putensen C. (2001). The Effects of Prone Positioning on Intraabdominal Pressure and Cardiovascular and Renal Function in Patients with Acute Lung Injury. Obstet. Anesthesia Dig..

[17] Danziger J.M., Chen K.P., Lee J., Feng M., Mark R.G., Celi L.A., Mukamal K.J. (2016). Obesity, Acute Kidney Injury, and Mortality in Critical Illness. Crit. Care Med..

[18] Griffiths M.J.D., McAuley D.F., Perkins G.D., Barrett N., Blackwood B., Boyle A., Chee N., Connolly B., Dark P., Finney S. (2019). Guidelines on the management of acute respiratory distress syndrome. BMJ Open Respir. Res..

[19] BMJ Open EPIdemiology of Surgery-Associated Acute Kidney Injury (EPIS-AKI): Study Protocol for a Multicentre, Observational Trial. https://bmjopen.bmj.com/content/11/12/e055705.

[20] Kellum J.A., Lameire N., Aspelin P., Barsoum R.S., Burdmann E.A., Goldstein S.L., Herzog C.A., Joannidis M., Kribben A., Levey A.S. (2012). Improving global outcomes (KDIGO) acute kidney injury work group. KDIGO clinical practice guideline for acute kidney injury. Kidney Int. Suppl..

[21] Waikar S.S., Bonventre J.V. (2009). Creatinine Kinetics and the Definition of Acute Kidney Injury. J. Am. Soc. Nephrol..

[22] Kellum J.A., Lameire N., on behalf of the KDIGO AKI Guideline Work Group (2013). Diagnosis, evaluation, and management of acute kidney injury: A KDIGO summary (Part 1). Crit. Care.

[23] Le Gall J.R., Lemeshow S., Saulnier F. (1993). A new Simplified Acute Physiology Score (SAPS II) based on a European/North American multicenter study. JAMA.

[24] Pourhoseingholi M.A., Vahedi M., Rahimzadeh M. (2013). Sample size calculation in medical studies. Gastroenterol. Hepatol. Bed Bench.

[25] Kirkpatrick A.W., Pelosi P., De Waele J.J., Malbrain M.L., Ball C.G., Meade M.O., Stelfox H.T., Laupland K.B. (2010). Clinical review: Intra-abdominal hypertension: Does it influence the physiology of prone ventilation?. Crit. Care.

[26] Sun J., Sun H., Sun Z., Yang X., Zhou S., Wei J. (2021). Intra-abdominal hypertension and increased acute kidney injury risk: A systematic review and meta-analysis. J. Int. Med Res..

[27] Magder S. (2015). Understanding central venous pressure: Not a Preload Index?. Curr. Opin. Crit. Care.

[28] Hamzaoui O., Teboul J.-L. (2022). Central venous pressure (CVP). Intensiv. Care Med..

[29] Chen X., Wang X., Honore P.M., Spapen H.D., Liu D. (2018). Renal failure in critically ill patients, beware of applying (central venous) pressure on the kidney. Ann. Intensiv. Care.

[30] Li D.-K., Wang X.-T., Liu D.-W. (2017). Association between elevated central venous pressure and outcomes in critically ill patients. Ann. Intensiv. Care.

[31] Chen C.-Y., Zhou Y., Wang P., Qi E.-Y., Gu W.-J. (2020). Elevated central venous pressure is associated with increased mortality and acute kidney injury in critically ill patients: A meta-analysis. Crit. Care.

[32] De Backer D., Aissaoui N., Cecconi M., Chew M.S., Denault A., Hajjar L., Hernandez G., Messina A., Myatra S.N., Ostermann M. (2022). How can assessing hemodynamics help to assess volume status?. Intensiv. Care Med..

[33] Wiedemann H.P., Wheeler A.P., Bernard G.R., Thompson B.T., Hayden D., Deboisblanc B., Connors A.F.J., Hite R.D., Harabin A.L., National Heart, Lung, and Blood Institute Acute Respiratory Distress Syndrome (ARDS) Clinical Trials Network (2006). Comparison of Two Fluid-Management Strategies in Acute Lung Injury. N. Engl. J. Med..

[34] Ahuja S., de Grooth H.-J., Paulus F., van der Ven F.L., Neto A.S., Schultz M.J., Tuinman P.R., van Akkeren J.P., Algera A.G., Algoe C.K. (2022). Association between early cumulative fluid balance and successful liberation from invasive ventilation in COVID-19 ARDS patients—Insights from the PRoVENT-COVID study: A national, multicenter, observational cohort analysis. Crit. Care.

[35] De Keulenaer B.L., De Waele J.J., Powell B., Malbrain M.L.N.G. (2009). What is normal intra-abdominal pressure and how is it affected by positioning, body mass and positive end-expiratory pressure?. Intensiv. Care Med..

[36] Jacobs R., Wise R.D., Myatchin I., Vanhonacker D., Minini A., Mekeirele M., Kirkpatrick A.W., Pereira B.M., Sugrue M., De Keulenaer B. (2022). Fluid Management, Intra-Abdominal Hypertension and the Abdominal Compartment Syndrome: A Narrative Review. Life.

[37] Wintjens M.S.J.N., van Rosmalen F., Hemmelder M.H., Hulsewe-Evers H.P.M.G., Kusters Y.H.A.M., Ubben J.F.H., van Renswouw D.A.M., Gilissen K.M.H., van der Horst I.C.C., van Mook W.N.K.A. (2023). Prone positioning is followed by a transient decrease in glomerular filtration rate: The prospective Maastricht Intensive Care COVID cohort. J. Nephrol..

[38] Scaramuzzo G., Gamberini L., Tonetti T., Zani G., Ottaviani I., Mazzoli C.A., Capozzi C., Giampalma E., Reggiani M.L.B., Bertellini E. (2021). Sustained oxygenation improvement after first prone positioning is associated with liberation from mechanical ventilation and mortality in critically ill COVID-19 patients: A cohort study. Ann. Intensiv. Care.

