# Supplementary material for: Incidence and Determinants of Acute Kidney Injury after Prone Positioning in Severe COVID-19 Acute Respiratory Distress Syndrome
Source: Healthcare (Basel). 2023 Nov 4;11(21):2903. doi: 10.3390/healthcare11212903 (PMC10647784; doi:10.3390/healthcare11212903)
Supplement: Supplementary file 1 [file healthcare-11-02903-s001.zip › healthcare-2610762-supplementary.pdf]

healthcare

Article

Incidence and Determinants of Acute Kidney Injury after Prone Positioning in Severe COVID-19 Acute Respiratory Distress Syndrome

Riccardo La Rosa [1], Benedetta Grechi [1], Riccardo Ragazzi [1,2], Valentina Alvisi [2], Giacomo Montanari [2], Elisabetta Marangoni [2], Carlo Alberto Volta [1,2], Savino Spadaro [1,2] and Gaetano Scaramuzzo [1,2,*]

1 Department of Translational Medicine and for Romagna, University of Ferrara, 44124 Ferrara, Italy; riccardo.larosa@edu.unife.it (R.L.R.); benedetta.grechi@edu.unife.it (B.G.); riccardo.ragazzi@unife.it (R.R.); vlc@unife.it (C.A.V.); savino.spadaro@unife.it (S.S.)
2 Anesthesia and Intensive Care Unit, Emergency Department, Azienda Ospedaliera Universitaria Ferrara, 44124 Ferrara, Italy; v.alvisi@ospfe.it (V.A.); g.montanari@ospfe.it (G.M.); e.marangoni@ospfe.it (E.M.)
* Correspondence: scrgtn@unife.it

Table S1. Multivariate regression analysis on predictors of Ventilator-free days (VFDs), censored at 28 days.

Variables	OR	95% C.I. for OR		p-value
		Lower	Upper	
Age, in years	-0.127	-0.326	0.099	0.290
BMI	-0.192	-0.596	0.037	0.082
**Previous story of CKD**	**-0.230**	**-17.899**	**-0.762**	**0.033**
SAPS before PP	-0.037	-0.325	0.238	0.759
PaO2/FiO2 ratio at admission	0.134	-0.008	0.044	0.173
Days of hospital before ICU	0.021	0.000	0.000	0.851
**Days of ICU before 1st PP cycle**	**-0.334**	**0.000**	**0.000**	**0.002**
Creatinine before PP	0.124	-1.745	6.266	0.265
**CVP before PP**	**0.271**	**0.123**	**0.936**	**0.011**
AKI development during the 1st PP	-0.015	-3.199	2.769	0.886
**Number of PP cycles**	**-0.308**	**-3.110**	**-0.698**	**0.002**
Constant	21.112			0.025

Data refers to the first pronation cycle for all patients. BMI = body mass index; CFB = cumulative fluid balance; SAPS = Simplified Acute Physiology Score; PP = prone positioning; CKD = chronic kidney disease; ICU = intensive care unit.

Table S2. Logistic regression analysis on predictors of ICU mortality.

Variables	OR	95% C.I. for OR		p-value
		Lower	Upper	
Age, in years	1.096	1.000	1.203	0.051
BMI	1.095	0.968	1.239	0.151
Previous story of CKD	0.000	0.000	0.000	0.999
SAPS before PP	1.048	0.944	1.163	0.383
PaO2/FiO2 ratio at admission	0.997	0.987	1.007	0.497
Days of hospital before ICU	1.000	1.000	1.000	0.557
**Days of ICU before 1st PP cycle**	**1.000**	**1.000**	**1.000**	**0.003**
Creatinine before PP	0.244	0.037	1.609	0.143
**CVP before PP**	**0.831**	**0.699**	**0.989**	**0.037**
AKI development during the 1st PP	0.468	0.134	1.637	0.235
**Number of PP cycles**	**2.823**	**1.417**	**5.622**	**0.003**
Constant	727363.6			0.051

Data refers to the first pronation cycle for all patients. BMI = body mass index; CFB = cumulative fluid balance; SAPS = Simplified Acute Physiology Score; PP = prone positioning; CKD = chronic kidney disease; ICU = intensive care unit.
